# Steric Effect of Antioxidant Diels-Alder-Type Adducts: A Comparison of Sanggenon C with Sanggenon D

**DOI:** 10.3390/molecules23102610

**Published:** 2018-10-11

**Authors:** Xican Li, Zhenxing Ren, Zimei Wu, Zhen Fu, Hong Xie, Langyu Deng, Xiaohua Jiang, Dongfeng Chen

**Affiliations:** 1 School of Chinese Herbal Medicine, Guangzhou University of Chinese Medicine, Guangzhou 510006, China; xiehongxh1@163.com (H.X.); dfd20061047@163.com (L.D.); 2 Innovative Research & Development Laboratory of TCM, Guangzhou 510006, China; 3 School of Basic Medical Science, Guangzhou University of Chinese Medicine, Guangzhou 510006, China; zhenxing2016@gmail.com (Z.R.); wuzimei_168@163.com (Z.W.); fuzhen@gzucm.edu.cn (Z.F.); 4 The Research Center of Basic Integrative Medicine, Guangzhou University of Chinese Medicine, Guangzhou 510006, China; 5 School of Biomedical Sciences, Faculty of Medicine, The Chinese University of Hong Kong, Sha Tin, Hong Kong 999077, China; xjiang@cuhk.edu.hk

**Keywords:** antioxidant, Diels-Alder-type adduct, sanggenon C, sanggenon D, steric effect

## Abstract

Sanggenons C and D are two Diels-Alder-type adducts from Chinese crude drug Sang-bai-pi. Structurally, both sanggenons construct stereoisomers. In the study, they were comparatively determined using four antioxidant assays, including ferric ion reducing antioxidant power (FRAP) assay, Cu^2+^-reducing assay, 1,1-diphenyl-2-picryl-hydrazl (DPPH•)-scavenging assay, and 2,2′-azino-bis (3-ethylbenzo-thiazoline-6-sulfonic acid radical (ABTS•^+^)-scavenging assay. Their Fe^2+^-binding reactions were explored using UV-Vis spectra. Finally, their cytoprotective effects were evaluated using flow cytometry. In electron transfer (ET)-based FRAP and Cu^2+^-reducing assays, sanggenon D was found to have lower IC_50_ values than sanggenon C; however, in multi-pathway-based DPPH•-scavenging and ABTS•^+^-scavenging assays, sanggenon C possessed lower IC_50_ values than sanggenon D. UV-Vis spectra suggested that sanggenon C generated a bathochromic-shift (286 nm → 302 nm) and displayed stronger UV absorption than sanggenon D. In flow cytometry, sanggenon C and sanggenon D, respectively, exhibited 31.1% and 42.0% early apoptosis-percentages towards oxidative-stressed mesenchymal stem cells (MSCs). In conclusion, both sanggenons may undergo multiple pathways (e.g., ET and Fe^2+^-binding) to protect MSCs against oxidative stress. In the mere ET aspect, sanggenon D possesses a higher level than sanggenon C, while in multi-pathway-based radical-scavenging, Fe^2+^-binding, and cytoprotection aspects, sanggenon C is more active than sanggenon D. These discrepancies can conclusively be attributed to the steric effect.

## 1. Introduction

**Figure 1:**
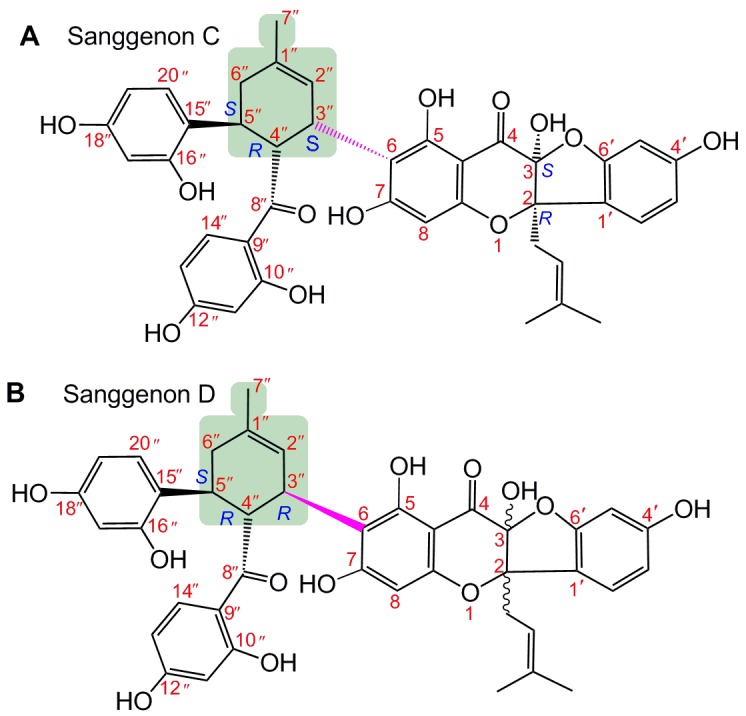
The structures of sanggenons C (**A**) and sanggenons D (**B**) (the Diels-Alder-type adduct skeletons are in light green).

Diels-Alder-type adducts are unusual natural products occurring in plants. Until now, about 60 Diels-Alder-type adducts have already been obtained from plants, especially from the *Morus* family [1]. Two earliest-found Diels-Alder-type adducts sanggenons C and D are typical examples (Figure 1) [2,3]. Recent studies indicated that sanggenon C had various bioactivities, such as anti-cancer [4,5], anti-inflammatory [6], and cytoprotective effects against hypoxia injury in cardiac cells [6]. To the best of our knowledge, no cutting-edge biotechnology, such as stem cell technology, has been used to study Diels-Alder-type adducts until now.

Stem cell technology is undoubtedly one of the most attractive therapeutic tools in recent decades. This technology brings hope to the treatment of human diseases through tissue transplantation and regeneration. As ideal seed cells of tissue transplantation and regeneration, mesenchymal stem cells (MSCs) are usually used for clinical or experimental studies, owing to several advantages (such as differentiation potential [7,8], easy access [9], expansion [9], and proliferation [10,11]).

However, in the process of MSC expansion and proliferation, oxidative stress greatly lowers their survival and limits their clinical applications [11]. The so-called oxidative stress is from various reactive oxygen species (ROS) or reactive nitrogen species (RNS), such as hydroxyl radicals (•OH), peroxide hydrogen molecules (H_2_O_2_), and nitric oxide (•NO). Particularly, when H_2_O_2_ encounters ferrous (Fe^2+^), it can undergo a Fenton reaction (Fe^2+^+ H_2_O_2_ → Fe^3+^+•OH + OH^−^) to give rise to hydroxyl radicals (•OH radicals) [12]. Hydroxyl radicals are known as the most powerful ROS form and can attack biomolecules (especially DNA [13]) to cause cellular damage [12]. Hence, supplementing phenolic Diels-Alder-type adducts is expected to resolve this bottleneck of cutting-edge stem cell technologies. As seen in Figure 1, both sanggenons C and D bear several phenolic –OHs and belong to phenolic Diels-Alder-type adducts. Thus, they are believed to act as antioxidants to protect MSCs from oxidative stress.

However, from the point of stereochemistry, two sanggenons are different: sanggenon C bears a (***2R***, ***3S***, ***3″S***, *4″R*, *5″S*) configuration, while sanggenon D displays a (***3″R***, *4″R*, *5″S*) configuration (Figure 1). The configurations of 2-C and 3-C in sanggenon D have remained unclear until now [14,15]. Nevertheless, sanggenons C and D are undoubtedly two steric isomers. In fact, a similar situation can also be observed in other Diels-Alder-type adducts, such as cathayanons A and B [16]. To our knowledge, no relevant study has discussed the possible steric effect in Diels-Alder-type adducts (and other phenolics). Thus, the present study tries to provide relevant evidence. The evidence is believed to be helpful to explain the steric effects in other Diels-Alder-type adducts, and to design new cytoprotector for stem cell technology.

## 2. Results and Discussion

In the study, FRAP assay has been fulfilled under pH 3.6. As seen in Figure S1A, the concentration dependency of sanggenons C and D increased the FRAP percentages. FRAP assay, however, is documented as a mere ET process [17,18], because such an acidic condition (pH 3.6) has remarkably suppressed H^+^ ionization. This implies that, when sanggenons C and D act as antioxidants, they may undergo ET pathways to exert their antioxidant abilities.

Unlike FRAP assay, Cu^2+^-reducing assay was conducted in pH 7.4 buffer, in which both sanggenons exhibited potent metal-reducing activity in a dose dependent manner (Figure S1B). It means that, under physiological pH 7.4, both sanggenons still have the ET potential. However, the ET potential of sanggenon D is higher than that of sanggenon C, according to the IC_50_ values (Table 1). Apparently, the difference in ET potential between sanggenons C and D can be attributed to the steric configuration of chiral atoms.

After all, antioxidation is not identical with a mere reducing reaction. In the antioxidant process, ET is usually accompanied by a proton (H^+^) transfer to give rise to a stable (semi) quinone form [13]. Detailed mechanisms are suggested to include hydrogen atom transfer (HAT) [19,20,21], sequential proton loss single electron transfer (SPLET) [22,23], proton coupled electron-transfer (PCET) [20,24], and sequential electron proton transfer (SEPT) [19,20]. For example, ABTS•^+^-scavenging, a single electron transfer reaction [25], has also been proven to be affected by H^+^ levels [26]. Thus, ABTS•^+^-scavenging is actually a multi-pathways-based antioxidant assay [18,27]. As seen in Figure S1C, sanggenons could dose-dependently increase the ABTS•^+^-scavenging percentages, suggesting that they could also undergo multiple pathways to exert the antioxidant action.

Evidence from DPPH•-scavenging assay further confirmed the above hypothesis based on ABTS•^+^-scavenging assay (Supplementary File S2D), because DPPH•-scavenging has also been demonstrated to occur via multi-pathways, including HAT [19,20,21], SPLET [22,23], SEPT [19,20], PCET [20,24], and ET [28,29]. However, the quantitative analysis of IC_50_ values (Table 1) indicated that, in multi-pathway-based ABTS•^+^-scavenging and DPPH•-scavenging aspects, sanggenon C is superior to its stereoisomer sanggenon D.

It should be noted that, some ROS can also be transformed through transition-metal (especially Fe^2+^) catalysis. For example, in the Fenton reaction, H_2_O_2_ molecules can be catalyzed by Fe^2+^ to produce •OH radicals. Therefore, the decrease of Fe^2+^ levels through a binding reaction can successfully reduce the •OH radicals to release cellular oxidative stress. Hence, iron binding by antioxidants has been developed to be an effective therapeutic approach for some oxidative stress diseases [30].

**Figure 2:**
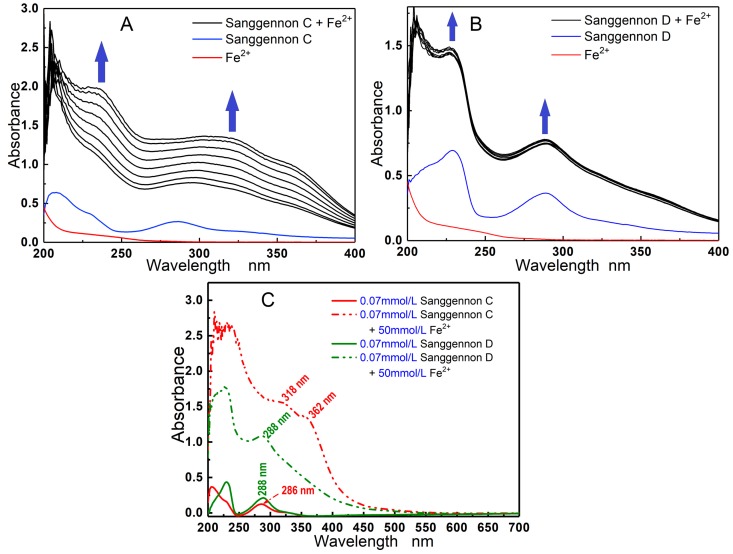
(**A**) UV spectra of reaction mixture of Fe^2+^ (50 mmol/L) with sanggenon C (0.07 mmol/L) within the initial 0–24 min; (**B**) UV spectra of reaction mixture of Fe^2+^ (50 mmol/L) with sanggenon D (0.07 mmol/L) within the initial 0–24 min; (**C**) the comparison of UV-vis spectra of sanggenon C and sanggenon D in Fe^2+^-binding reaction at 24 min.

In the present study, two stereoisomers sanggenons C and D were found to be able to bind Fe^2+^. As illustrated in Figure 2A,B, within the initial 3 min, sanggenons C and D presented similar UV-peak intensities; however, in the subsequent 20 min, sanggenon C continued to increase the UV-peak intensity, while sanggenon D basically stopped the tendency. At the last stage of scanning (at 24 min), the UV peaks of sanggenon C were generally higher than those of sanggenon D. In the aspect of peak site, sanggenon C apparently produced a bathochromic shift (286 nm → 302 nm), and sanggenon D gave no bathochromic shift (289 nm → 289 nm). The data of UV-peak intensity and bathochromic shift showed that sanggenon C possessed stronger Fe^2+^-binding ability than sanggenon D.

The above difference can only be attributed to the steric configurations. As seen in Figure 1, in sanggenon C, 3″-substituent group is on one side together with 4″-substituent group. It provides a possibility that 5-OH, 7-OH, 8″-carbonyl group, and 10″-OH jointly bind Fe^2+^ to form a big cyclic complex. However, in sanggenon D, 3″-substituent group is on the reverse side with 4″-substituent group. It is impossible for the above phenolic -OH and carbonyl groups to jointly bind Fe^2+^ to form a big cyclic complex. As a result, sanggenon C is more active than sanggenon D in the Fe^2+^-binding reaction.

As mentioned above, the steric configurations of 2-C and 3-C atoms in sanggenon D are unknown. If the steric configurations of 2-C and 3-C atoms in sanggenon D are identical with those in sanggenon C; The difference in Fe^2+^-binding reaction can solely be attributed to the steric configuration of 3″-C. However, if the steric configurations of 2-C and 3-C atoms in sanggenon D are not identical with those in sanggenon C. The Fe^2+^-binding difference can be attributed to the steric configurations of 3″-C, 2-C, and 3-C atoms.

**Figure 3:**
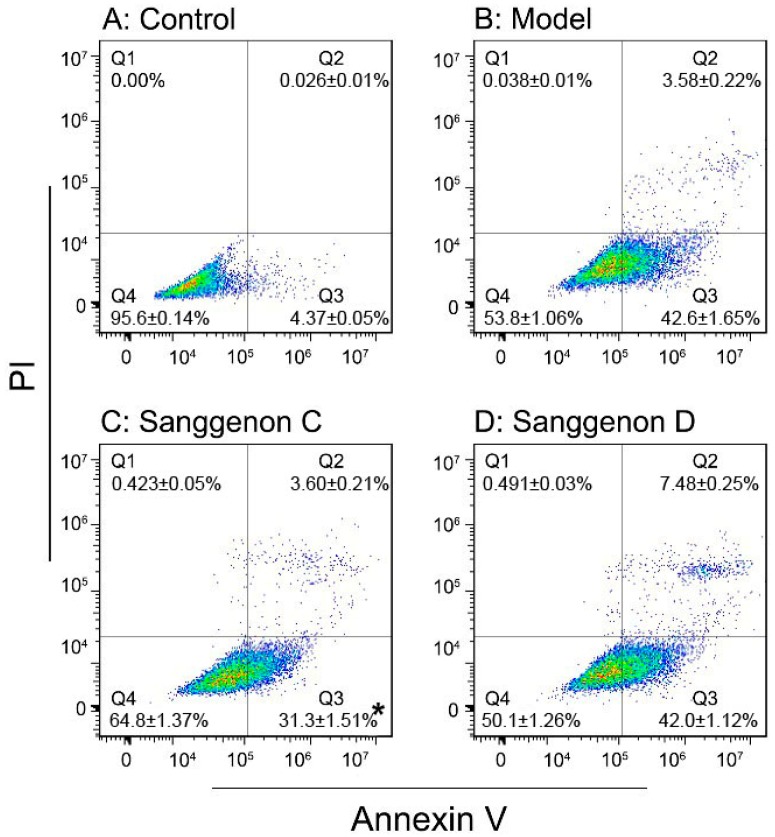
Cytoprotective effect of sanggenons C and D towards MSCs under oxidative stress based on flow cytometry assay (left upper, control; right upper, model; left lower, sanggenon C; right lower, sanggenon D). (MSCs, bone marrow-derived mesenchymal stem cells; each value is expressed as the mean ± SD, n = 3; * *p* < 0.05 vs. model.).

As seen in Figure 1, 2-C and 3-C atoms are far from the Fe^2+^-binding reaction center; their effects may be very limited in Fe^2+^-binding reaction. 

In short, the difference in the Fe^2+^-binding reaction is seemingly from the difference in steric configuration of chiral atoms (especially 3″-C); however, the steric configurations (*R/S*) can change the chemical environment of these huge substituent groups and then affect molecular reactivity (especially metal-binding). This may be caused by the steric effect, which is usually ignored by chemists and pharmacists.

The steric effect can lead to different cytoprotective effects. As indicated by flow cytometry assay (Figure 3), the MSCs in the control, model, sanggenon C, and sanggenon D groups exhibited 4.37 ± 0.05%, 42.6 ± 1.65%, 31.3 ± 1.51%, and 42.0 ± 1.12% early apoptosis percentages, respectively. The results revealed that sanggenon C could effectively inhibit apoptosis of MSCs under oxidative stress and protect them from oxidative stress. However, sanggenon D was basically inactive. The difference from the cellular model is roughly parallel to those from multi-pathway-based radical-scavenging and Fe^2+^-binding assays.

## 3. Materials and Methods

### 3.1. Animals and Chemicals

Sprague-Dawley (SD) rats of 4 weeks of age were obtained from the Animal Center of Guangzhou University of Chinese Medicine. The protocol of this experiment was performed under the supervision of the Institutional Animal Ethics Committee in Guangzhou University of Chinese. Sanggenon C (CAS number: 80651-76-9, C_40_H_36_O_12_, M.W. 708.7, 98%, Supplementary File S2) and sanggenon D (CAS number: 81422-93-7, C_40_H_36_O_12_, M.W. 708.7, 97%) were obtained from Chengdu Biopurify Phytochemicals Ltd. (Chengdu, China, Supplementary File S3). The 1,1-diphenyl-2-picryl-hydrazl radical (DPPH•), (±)-6-hydroxyl-2,5,7,8-tetramethlychromane-2-carboxylic acid (Trolox), 2,9-dimethyl-1,10-phenanthroline (neocuproine), and 2,4,6-tripyridyltriazine (TPTZ) were purchased from Sigma-Aldrich Shanghai Trading Co. (Shanghai, China). (NH_4_)_2_ABTS [2,2′-azino-bis (3-ethylbenzo-thiazoline-6-sulfonic acid diammonium salt)] was purchased from Amresco Chemical Co. (Solon, OH, USA). Fetal bovine serum (FBS), Dulbecco’s modified Eagle’s medium (DMEM), and trypsin were from Gibco (Grand Island, NY, USA). Annexin V/propidium iodide (PI) assay kit was obtained from Abcam (Cambridge, UK). All other reagents were of analytical grade.

### 3.2. FRAP Assay

The FRAP assay was carried out according to the method of Benzie and Strain [17,31]. Briefly, the FRAP reagent was prepared freshly by mixing 10 mM TPTZ, 20 mM FeCl_3_, and 0.25 M acetate buffer at 1:1:10 at pH 3.6. The test sample (x = 2–10 μL, 0.5 mg/mL) was added to (20 − x) μL of 95% ethanol followed by 80 μL of FRAP reagent. The absorbance was measured at 595 nm after a 15 min incubation at room temperatures using distilled water as the blank. The relative reducing power of the sample was calculated using the formula:

(1) $$Relative\;reducing\;effect\;\%\;=\frac{A-A_{min}}{A_{max}-A_{min}}\times 100\%$$

in which *A_max_* is the maximum absorbance at 595 nm and *A_min_* is the minimum absorbance at 595 nm in the test. *A* is the absorbance at 595 nm of sample.

### 3.3. Cu^2+^-Reducing Power Assay

The Cu^2+^-reducing power assay was performed based on a previously published method [19]. Briefly, 12 μL of CuSO_4_ aqueous solution (10 mmol/L), 12 μL of neocuproine ethanolic solution (7.5 mmol/L), and (75 − x) μL of NH_4_OAc buffer solution (0.1 mol/L, pH 7.5) were added to wells with different volumes of sample (0.1 mg/mL, 2–10 μL). The absorbance at 450 nm after 30 min was determined on a microplate reader (Multiskan FC, Thermo Scientific, Shanghai, China). The relative Cu^2+^-reducing power was calculated using the formula described above in Section 3.2

### 3.4. ABTS•^+^-Scavenging Assay

The ABTS•^+^-scavenging activity was assessed according to the method [32,33]. The ABTS• ^+^ was produced by mixing 0.2 mL of (NH_4_)_2_ABTS (7.4 mmol/L) with 0.35 mL of K_2_S_2_O_8_ (2.6 mmol/L). The mixture was kept in the dark at room temperature for 12 h and then diluted with distilled water (about 1:20), so that its absorbance at 734 nm was measured on a microplate reader (Multiskan FC, Thermo Scientific, Shanghai, China). To determine the scavenging activity, the test sample (x = 2–10 μL, 0.1 mg/mL) was added to (20 − x) μL of distilled water followed by 80 μL of ABTS• ^+^ reagent, and the absorbance at 734 nm was measured 3 min after the initial mixing, using distilled water as the blank. The percentage inhibition of the samples was calculated as follows:

(2) $$Scavenging\;\%\;=\frac{A_{0}-A}{A_{0}}\times 100\%$$

in which *A*_0_ indicates the absorbance of the blank and *A* indicates the absorbance of the sample, sanggenons C and D.

### 3.5. DPPH•-Scavenging Assay

DPPH• radical-scavenging activity was determined as previously described [34]. Briefly, 80μL of DPPH• solution (0.1 mol/L) was mixed with the indicated concentrations of sample (0.5 mg/mL, 4–20 μL) dissolved in methanol. The mixture was maintained at room temperature for 30 min, and the absorbance was measured at 519 nm on a microplate reader (Multiskan FC, Thermo Scientific, Shanghai, China). The DPPH• scavenging percentage was calculated based on the formula presented in Section 3.4.

### 3.6. UV-Vis-Spectra Analysis of Fe^2+^-Binding With Sanggenons

The sanggenon-Fe^2+^ complex was evaluated by UV-Vis-spectroscopy method [35]. For these experiments, 300 μL of a methanolic solution of sanggenons C and D (1 mg/mL) and 100 μL of an aqueous solution of FeCl_2_·4H_2_O (100 mg/mL) was added to 600 μL of an aqueous of the mixture of distilled water and methanol (1:1). The solution was then mixed vigorously. Subsequently, the mixture was diluted 20-fold, and the spectrum was obtained using a UV-Vis spectrophotometer every 3 min (Unico 2600A, Shanghai, China) from 200–400 nm.

### 3.7. Flow Cytometry Assay

The MSCs were cultured according to our previous report with slight modifications [10,36]. In brief, bone marrow was obtained from the femur and tibia of rat. The marrow samples were diluted with DMEM (LG: low glucose) containing 10% FBS. MSCs were prepared by gradient centrifugation at 900 g for 30 min on 1.073 g/mL Percoll. The prepared cells were detached by treatment with 0.25% trypsin and passenged into cultural flasks at the density of 1 × 10^4^/cm^2^. The MSCs at passage 3 were evaluated for cultured cell homogeneity using detection of CD44 by flow cytometry and used for the investigation. The prepared cells were detached by treatment with 0.25% trypsin and passaged in culture flasks at the density of 1 × 10^4^/cm^2^. In the control group, MSCs were incubated in DMEM. In the model and sample groups, MSCs were treated with H_2_O_2_ (200 μM). After incubation for 3 h, MSCs in the control group and model group were incubated for 24 h in DMEM, while MSCs in the sample groups were incubated for 24 h in DMEM with sanggenons C and D.

The cells were then analyzed using flow cytometry assay described as the literature [37]. Briefly, the cultured cells were harvested by trypsin (0.05%) digestion in phosphate-buffered saline (PBS). The cells (5 × 10^5^/cm^2^) were collected by centrifugation, then re-suspended cells in 500 μL of 1X Binding Buffer; at the same time, 5 μL of Annexin V-FITC and 5 μL of propidium iodide were added. After incubating at room temperature for 5 min in the dark, fluorescence was measured using flow cytometry (Accuri C6, BD, USA) with standard software.

### 3.8. Statistical Analysis

The analysis was made in triplicate and the data were recorded as mean ± SD (standard deviation). The dose response curves were plotted using Origin 8.0 professional software (Origin Lab, Northampton, MA, USA). The IC_50_ value was defined as the final concentration of 50% radical inhibition (or relative metal-reducing power). Statistical comparisons were made by one-way ANOVA to detect significant difference using SPSS 13.0 (SPSS Inc., Chicago, IL, USA) for windows. *p* < 0.05 was considered statistically significant.

## 4. Conclusions

As two Diels-Alder-type adduct stereoisomers, sanggenons C and D may undergo an antioxidant approach to protect MSCs from oxidative stress. However, in the mere ET potential, sanggenon D is more effective than sanggenon C. In multi-pathway-based radical-scavenging, Fe^2+^-binding, and cytoprotection potentials, sanggenon C is more effective than sanggenon D. These differences can be attributed to the steric configurations from chiral atoms (especially 3″-C).

## Figures and Tables

**Table 1:** The IC_50_ values of sanggenons C and D in various antioxidant assays.

Assays	Sanggenon C μg/mL (μM)	Sanggenon D μg/mL (μM)	Trolox µg/mL (μM)
FRAP	285.2 ± 18.3 (402.5 ± 25.8 ^c^)	151.4 ± 21.2 (213.7 ± 30.0 ^b^)	20.8 ± 1.4 (83.1 ± 5.5 ^a^)
Cu^2+^-reducing	7.6 ± 0.2 (10.8 ± 0.3 ^b^)	5.8 ± 0.0 (8.2 ± 0.0 ^a^)	5.7 ± 0.0 (22.8 ± 0.0 ^c^)
ABTS•^+^-scavenging	3.8 ± 0.3 (5.4 ± 0.4 ^a^)	7.2 ± 0.5 (10.2 ± 0.7 ^b^)	5.9 ± 0.1 (23.7 ± 0.2 ^c^)
DPPH•-scavenging	81.4 ± 1.9 (114.9 ± 2.7 ^b^)	104.0 ± 1.2 (146.8 ± 1.7 ^c^)	4.8 ± 0.1 (19.4 ± 0.6 ^a^)

The IC_50_ value (in μg/mL unit) refers to the final concentration of 50% radical inhibition or relative metal-reducing power. It was calculated by linear regression analysis, which was conducted by Origin 8.0 professional software and expressed as the mean ± SD (n = 3). The IC_50_ value (μg/mL) was conversed into μM and collected in brackets. The IC_50_ values in μM with different superscripts (a or b) in the same row are significantly different (*p* < 0.05). Trolox is the positive control.

## Supplementary Materials

Supplementary materials are available online. Supplementary File S1: Dose response curves of FRAP, Cu^2+^ reducing, ABTS, and DPPH assays, Supplementary File S2: Certificate of analysis of sanggenon C; Supplementary File S3: Certificate of analysis of sanggenon D.

## Abbreviations

The following abbreviations are used in this manuscript:

ABTS	2,2′-azino-bis(3-ethylbenzo-thiazoline-6-sulfonic acid diammonium) salt
bmMSCs	bone marrow-derived mesenchymal stem cells
DMEM	Dulbecco’s modified Eagle’s medium
DPPH	Dulbecco’s modified Eagle’s medium
ET	electron transfer
FBS	fetal bovine serum
FRAP	ferric reducing antioxidant power
HAT	hydrogen atom transfer
PCET	proton-coupled electron transfer
ROS	reactive oxygen species
RNS	reactive nitrogen species
SD	standard deviation
SEPT	sequential electron proton transfer
SPLET	sequential proton loss single electron transfer
TPTZ	2,4,6-tris (2-pyridyl-s-triazine)
Trolox	(±)-6-hydroxyl-2,5,7,8-tetramethlychromane-2-carboxylic acid
