# Supplementary material for: Steric Effect of Antioxidant Diels-Alder-Type Adducts: A Comparison of Sanggenon C with Sanggenon D
Source: Molecules. 2018 Oct 11;23(10):2610. doi: 10.3390/molecules23102610 (PMC6222520; doi:10.3390/molecules23102610)
Supplement: Supplementary file 1 [file molecules-23-02610-s001.zip › Suppl. 3 Certificate Sanggenon D.pdf]

成都普瑞法科技开发有限公司
Chengdu Biopurify Phytochemicals Ltd.

文件编码（DOC NO.）：PRF-COA-BP125201

产品分析证书
Certificate of Analysis

中文名称：桑根酮D

English Name：Sanggenone D
别名(Alias):

产品编码(Cat. No.):BP1252
CAS Number: 81422-93-7
分子式(M. F.): C40H36O12
分子量(M. W.): 708.716

批号(Batch No.): PRF7110422
报告日期(Report date): 2016/11/4

Structure

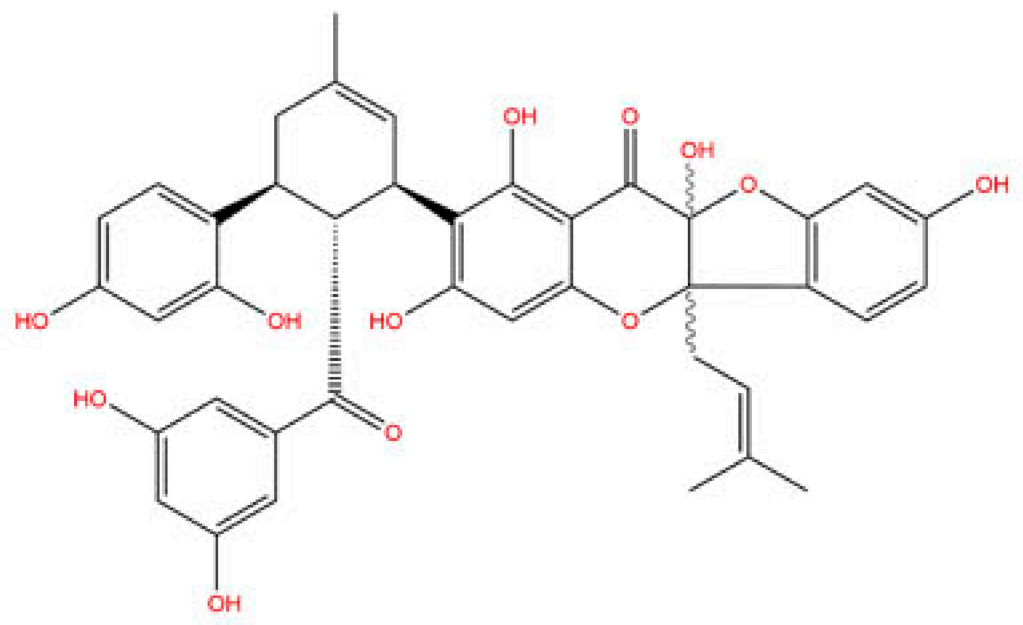

检验结果（Analytical result）：

检验项目（Test Item）	检验指标（Specifications）	检验结果（Results）
外观Appearance	Soil yellow powder	Soil yellow powder
干燥失重Loss on drying	＜3.0%	1.28%
纯度Purity (HPLC-DAD, 286nm)*	≥97.0%	97.19%
质谱Mass	708.716±1	Conforms
核磁NMR	Comply with the structure	Conforms

*** 色谱图见附件（Please find HPLC chromatography attached.）**

贮存条件（Storage）:2~8℃

复测期（Retest date）:two years (2018-11-03) under conditions list above.

备注（Remarks）：如遇质量问题，请于收到产品之日起 15 日内与我们联系。

In case of quality issue, please contact us within 15 days after receipt of the product.

QC: Zhang Ling   QA: Wu Qi

Date: 2016年11月4日   Date: 2016年11月4日

Chengdu Biopurify Phytochemicals Ltd. 成都普瑞法科技开发有限公司 质检专用章 Quality Approval Seal

Tel：+86-28-82633987   Fax：+86-28-82633165

http://www.biopurify.com   Email：sales@biopurify.com   biopurify@gmail.com

Empower 2 software

Chengdu Biopurify Phytochemicals Ltd.

SAMPLE INFORMATION

Sample Name:	Kuwanon D PRF7110422	Acquired By:	System
Sample Type:	Unknown	Sample Set Name:	
Vial:	70	Acq. Method Set:	Kuwanon D
Injection #:	1	Processing Method:	Samples
Injection Volume:	10.00 ul	Channel Name:	286.0nm
Run Time:	25.0 Minutes	Proc. Chnl. Descr.:	PDA 286.0 nm
Date Acquired:	2016-11-4 11:00:56 CST		
Date Processed:	2016-11-4 12:38:16 CST		

Auto-Scaled Chromatogram

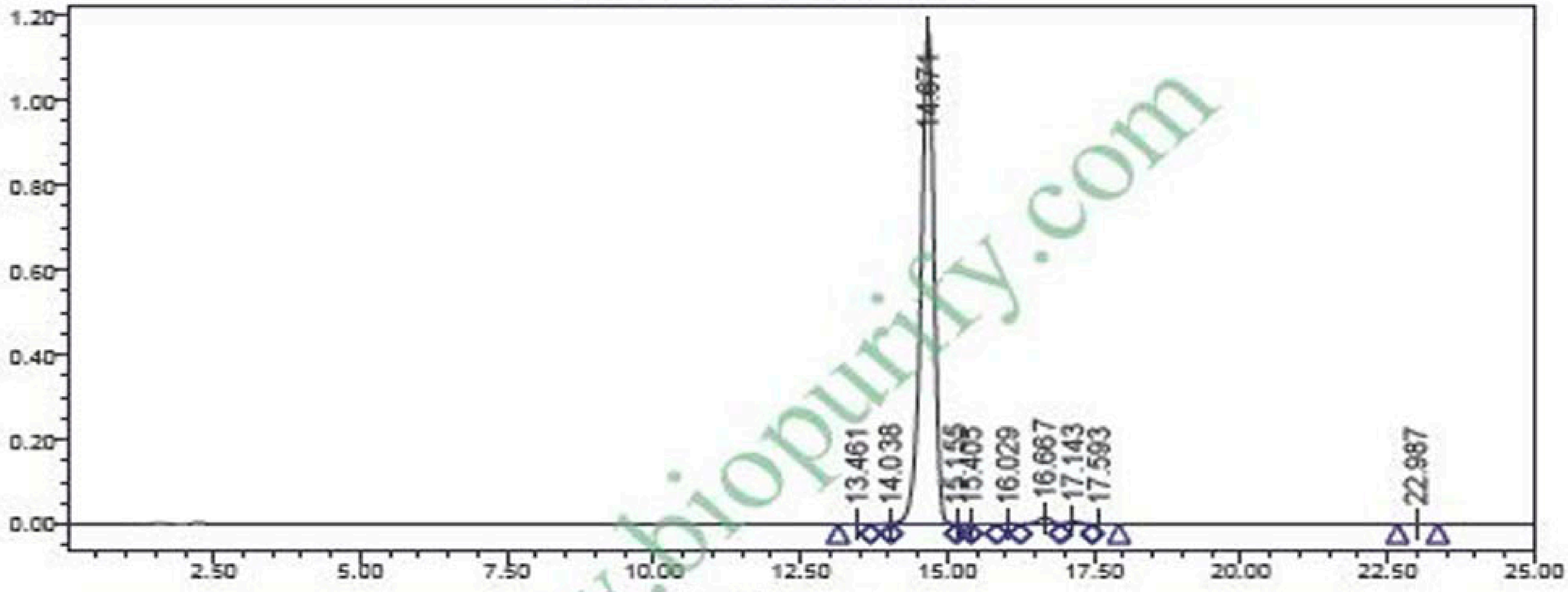

Peak Results

	RT	Area	% Area	USP Plate Count	USP Resolution
1	13.461	11555	0.06	24456.42	
2	14.038	22422	0.12		
3	14.671	17828454	97.19	21881.34	
4	15.155	25663	0.14		
5	15.405	27899	0.15		
6	16.029	26536	0.14		
7	16.667	264302	1.44	23818.92	
8	17.143	113600	0.62	23682.18	1.06
9	17.593	12254	0.07		
10	22.987	11883	0.06	62181.31	

www.biopurify.com

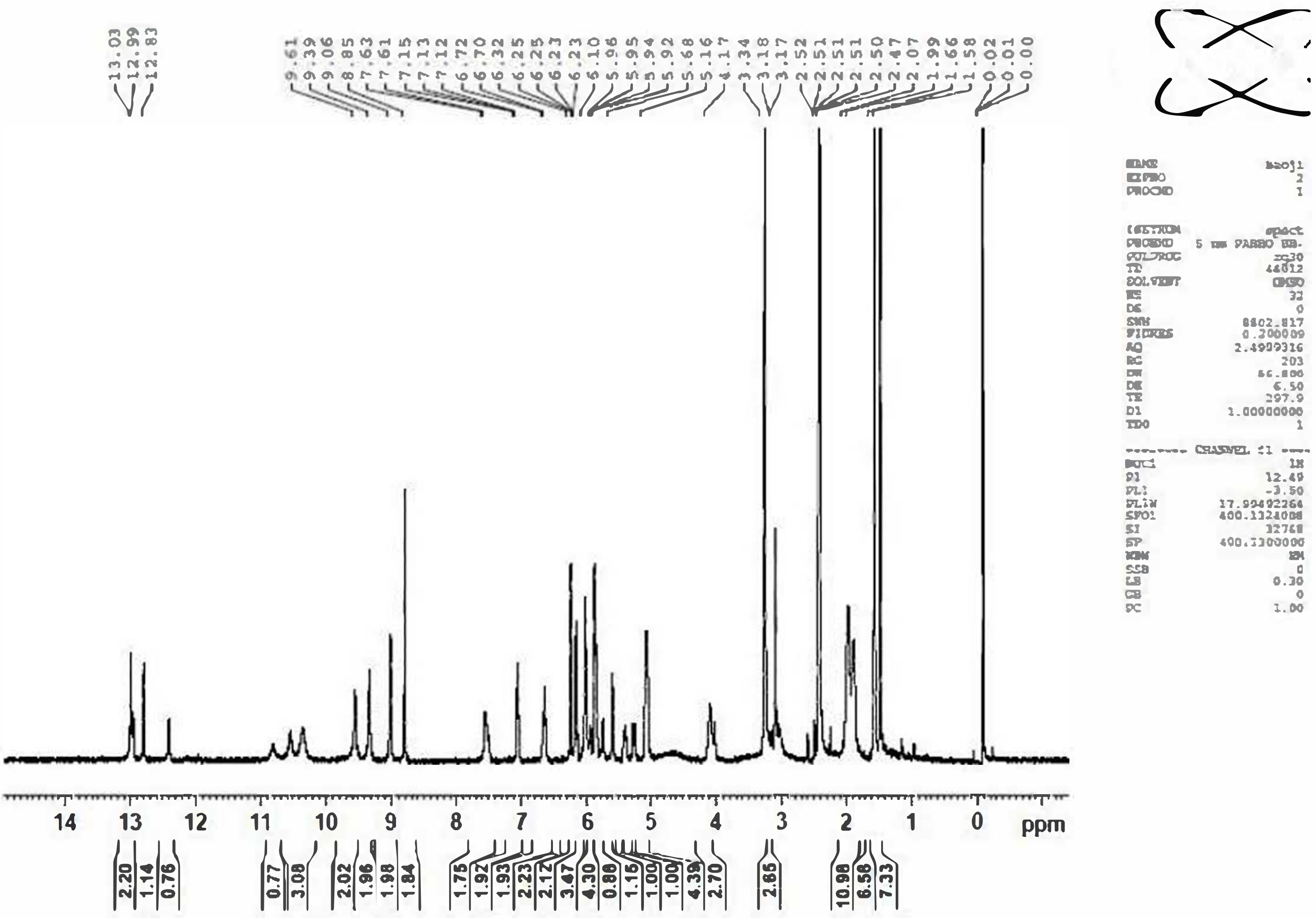

13.03 12.99 12.83
9.61 9.39 9.06 8.85 7.63 7.61 7.15 7.13 7.12 6.72 6.70 6.32 6.25 6.25 6.23 6.23 6.10 5.96 5.95 5.94 5.92 5.68 5.16 4.17 3.34 3.18 3.17 2.52 2.51 2.51 2.51 2.50 2.47 2.07 1.99 1.66 1.58 0.02 0.01 0.00
14 13 12 11 10 9 8 7 6 5 4 3 2 1 0 ppm
2.20 1.14 0.76 0.77 3.08 2.02 1.96 1.98 1.84 1.75 1.92 1.93 2.23 2.12 3.47 4.30 0.88 1.15 1.00 1.00 4.39 2.70 2.65 10.98 6.58 7.33
NAME baoji
EXPNO 2
PROCNO 1
SOLVENT DMSO
NS 32
DS 0
RG 203
D1 1.00000000
TD0 1
CHANNEL f1
NUC1 1H
SI 32768
SF 400.1300000
WDW EM
SSB 0
LB 0.30
GB 0
PC 1.00
