# Supplementary material for: Steric Effect of Antioxidant Diels-Alder-Type Adducts: A Comparison of Sanggenon C with Sanggenon D
Source: Molecules. 2018 Oct 11;23(10):2610. doi: 10.3390/molecules23102610 (PMC6222520; doi:10.3390/molecules23102610)
Supplement: Supplementary file 1 [file molecules-23-02610-s001.zip › Suppl. 1 Dose response curves.pdf]

Supplemental Material-1

Sanggenons C and D as Antioxidant Diels-Alder-Type Adducts Protecting Mesenchymal Stem Cells from Oxidative Stress: Evidence of the Steric Effect

Xican Li [1, 2, *,†], Zhenxing Ren [3, 4,†], Zimei Wu [3, 4], Zhen Fu [3, 4], Hong Xie [1], Langyu Deng [1], Xiaohua Jiang [5], Dongfeng Chen [3, 4, *]

[1] School of Chinese Herbal Medicine, Guangzhou University of Chinese Medicine, Guangzhou 510006, China; lixc@gzucm.edu.cn (X.L.); xiehongxh1@163.com (H.X.); dly9001@qq.com (L.D.)
[2] Innovative Research & Development Laboratory of TCM, Guangzhou 510006, China;
[3] School of Basic Medical Science, Guangzhou University of Chinese Medicine, Guangzhou 510006, China; 251351061@qq.com (Z.R.); 1244642557@qq.com (Z.W.); fuzhen@gzucm.edu.cn (Z.F.); chen888@gzucm.edu.cn (D.C.)
[4] The Research Center of Basic Integrative Medicine, Guangzhou University of Chinese Medicine, Guangzhou 510006, China.
[5] School of Biomedical Sciences, Faculty of Medicine, The Chinese University of Hong Kong, Sha Tin, Hong Kong 999077, China; xjiang@cuhk.edu.hk (X.J.)

*Corresponding author. E-mail: lixc@gzucm.edu.cn (X.L.); chen888@gzucm.edu.cn (D.C.). **Tel**: +86-20-39358076

†Equal contributors

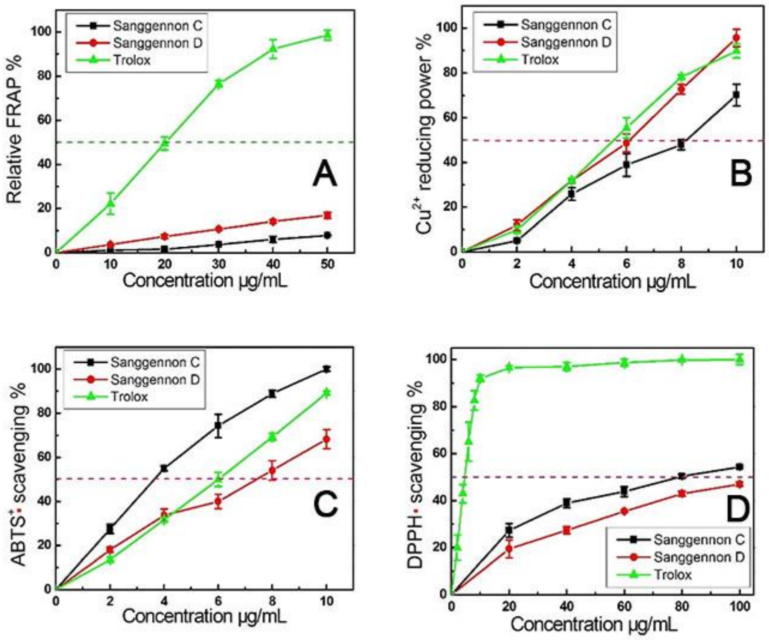

Suppl. 1 The dose-response curves of sanggenon C &D in various antioxidant assays: (A) FRAP assay; (B) Cu-reducing power assay; (C) ABTS•$^{+}$ scavenging assay; (D) DPPH• scavenging assay. (Each value is expressed as mean±SD, n=3)
