# Supplementary material for: Steric Effect of Antioxidant Diels-Alder-Type Adducts: A Comparison of Sanggenon C with Sanggenon D
Source: Molecules. 2018 Oct 11;23(10):2610. doi: 10.3390/molecules23102610 (PMC6222520; doi:10.3390/molecules23102610)

成都普瑞法科技开发有限公司
Chengdu Biopurify Phytochemicals Ltd.

文件编码（DOC NO.）：**PRF-COA-BP125101**

产品分析证书
Certificate of Analysis

中文名称：桑根酮C

English Name：Sanggenon C
别名(Alias):

产品编码(Cat. No.):BP1251
CAS Number: 80651-76-9
分子式(M. F.): C40H36O12
分子量(M. W.): 708.716

批号(Batch No.): PRF8041321
报告日期(Report date): 2017-04-13

Structure

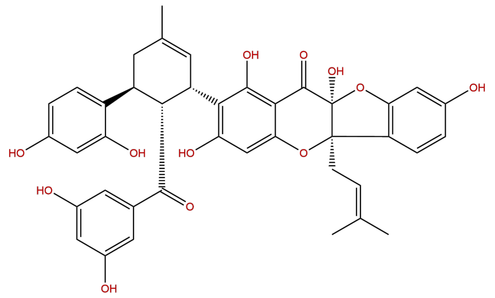

检验结果（Analytical result）：

**检验项目（Test Item）**	**检验指标（Specifications）**	**检验结果（Results）**
外观Appearance	Tan powder	Tan powder
干燥失重Loss on drying	＜3.0%	1.18%
纯度Purity (HPLC-DAD,286nm)*	≥98.0%	98.28%
质谱Mass	708.7±1	Conforms
核磁NMR	Comply with the structure	Conforms

*** 色谱图见附件（Please find HPLC chromatography attached.）**

检测方法（Test Method）： Column: Thermo HyPURITY C18，4.6*150mm，3.0um; **Column temperature**: 30℃; **Detection Mode:** UV286nm;

Flow Rate: 1.0ml/min; **Sample dissolution:** Methanol; **Mobile Phase: A**, 0.1% Phosphoric acid in water **B,** Acetonitrile; **Gradient elution:** B, 35%-50%,15min.

贮存条件（Storage）:2~8℃, protected from light, keep package airproofed when not in use.

复测期（Retest date）:two years (2019-04-12) under conditions list above.

QC: Zhang Ling　　　　QA: Wu Qi

Date: 2017-04-13　　　　Date: 2017-04-13

备注(Remarks): The sample solutions should be prepared and used on the same day, it is the best preparing the solutions immediately before use. If the solutons have to be made up in advance, it should be made as aliquots in tightly sealed vials at less than -20℃. Generally, these might be useable for up to two weeks.
In case of quality issue, please contact us within 15 days after receipt of the product.

Tel：+86-28-82633397　　Fax：+86-28-82633165

http://www.phytopurify.com　　Email：sales@biopurify.com　　biopurify@gmail.com

Chengdu Biopurify Phytochemicals Ltd.

SAMPLE INFORMATION

Sample Name:	Sanggenon C	Acquired By:	System
Sample Type:	Unknown	Sample Set Name:	
Vial:	79	Acq. Method Set:	Sanggenon C
Injection #:	1	Processing Method:	Samples
Injection Volume:	5.00 ul	Channel Name:	286.0nm
Run Time:	25.0 Minutes	Proc. Chnl. Descr.:	PDA 286.0 nm
Date Acquired:	2017-4-13 13:36:57 CST		
Date Processed:	2017-4-13 14:21:03 CST		

Auto-Scaled Chromatogram

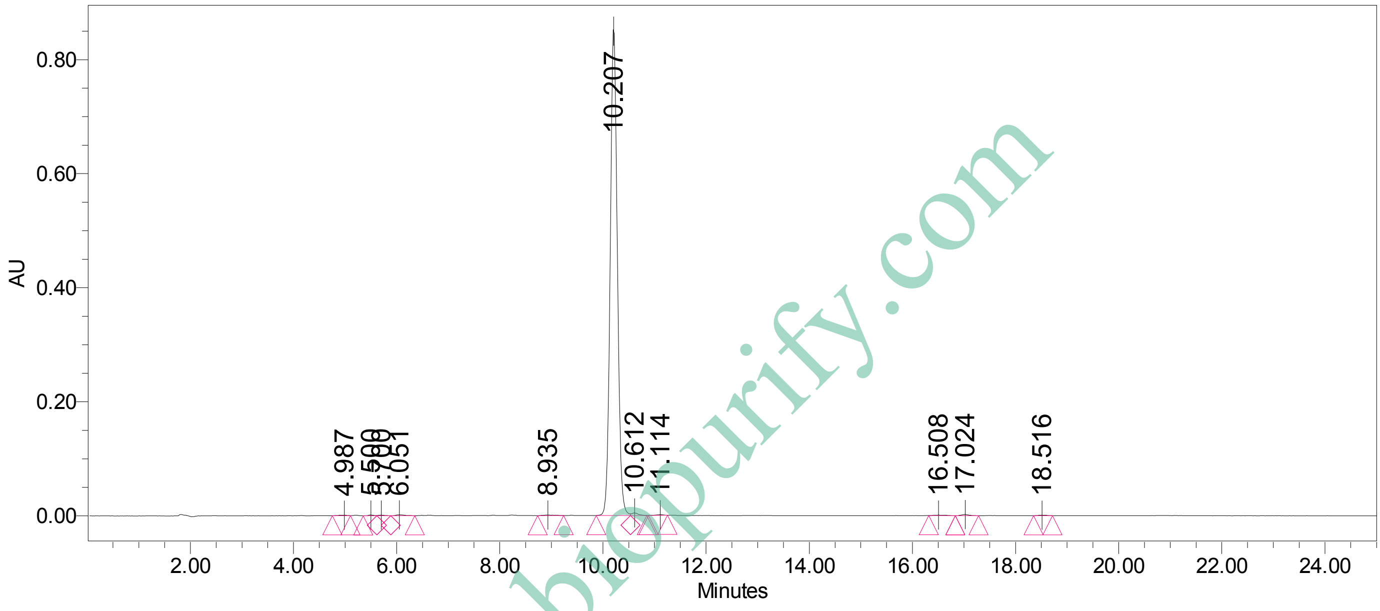

www.biopurify.com

Peak Results

	Name	RT	Area	% Area	USP Plate Count	USP Resolution
1		4.987	9160	0.11	4367.3	
2		5.500	9127	0.11	14729.7	1.2
3		5.700	8033	0.10	6709.6	0.8
4		6.051	15873	0.19	9614.8	1.3
5		8.935	12993	0.16	10000.3	9.3
6		10.207	8184191	98.28	26217.7	4.4
7		10.612	36977	0.44		1.6
8		11.114	9788	0.12	25689.7	2.1
9		16.508	12318	0.15	4938.7	15.7
10		17.024	20343	0.24	81329.3	1.4
11		18.516	8353	0.10	76093.1	4.6

NMR：

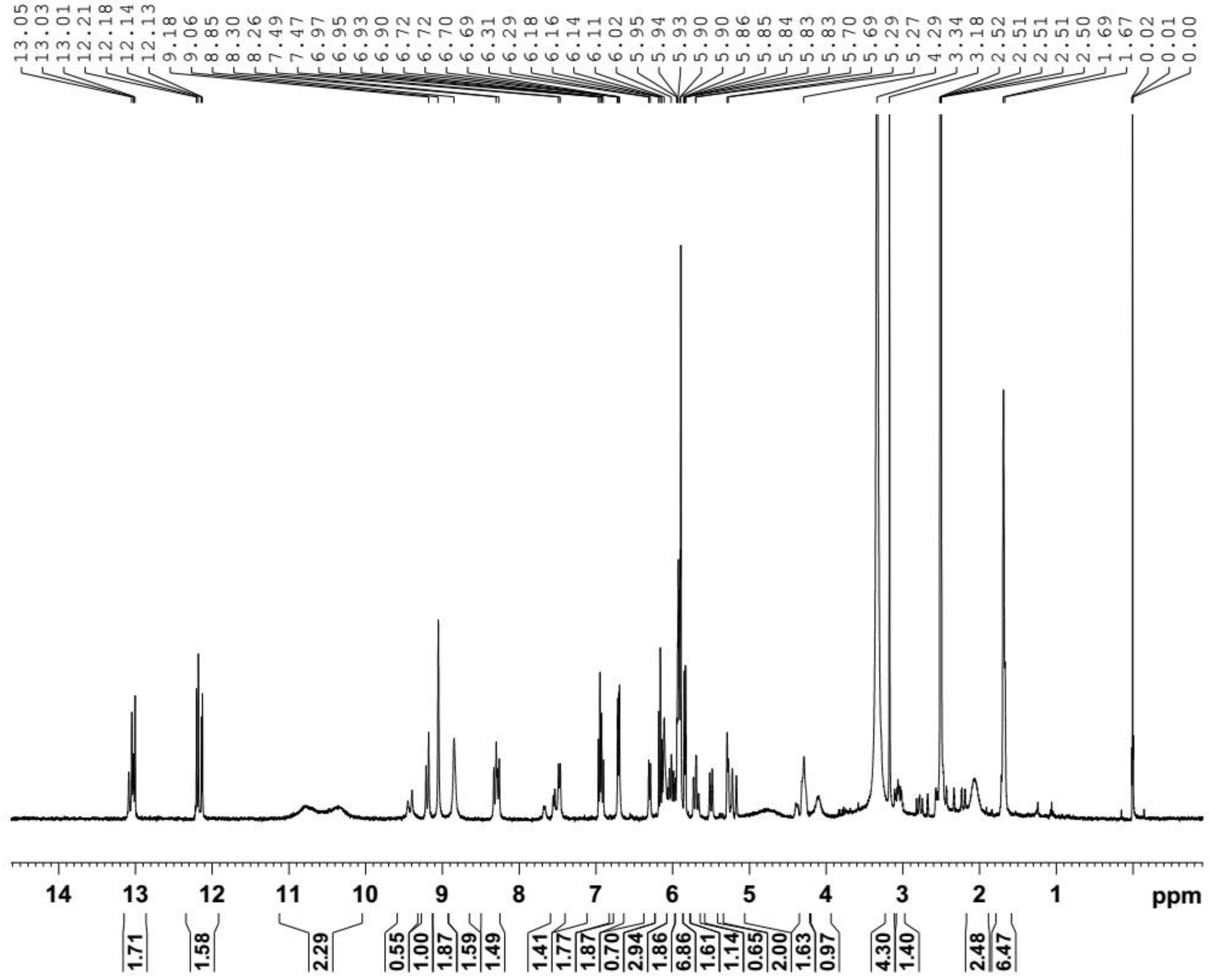

Supplement: Supplementary file 1 [file molecules-23-02610-s001.zip › Suppl. 2 Certificate Sanggenon C.pdf]
